# Supplementary material for: Screening de novo designed protein binders in unpurified lysate using flow induced dispersion analysis
Source: Protein Sci. 2025 Sep 13;34(10):e70286. doi: 10.1002/pro.70286 (PMC12432402; doi:10.1002/pro.70286)
Supplement: Supplementary file 1 — FIGURE S1: Purification of ALFA‐tag binders. Representative SDS–PAGE illustrating the Ni‐NTA purification of ALFA‐tag binders. FIGURE S2: SDS–PAGE analysis of soluble and insoluble fractions after chemical lysis for ALFA‐tag binders. E. coli cells expressing binders 2B‐5B were lysed with BPER reagent and the proteins present in the soluble (Sol.) and insoluble (Insol.) fractions visualized by SDS–PAGE. Cells with no expression of the protein (Neg.) and a sample of purified protein (Pure) were loaded for comparison. The soluble fractions were used for FIDA measurements. FIGURE S3: SDS–PAGE analysis of soluble and insoluble fractions after heat lysis for ALFA‐tag binders. E. coli cells expressing binders 2B‐5B were subjected to heat lysis and the proteins present in the soluble (Sol.) and insoluble (Insol.) fractions visualized by SDS–PAGE. Cells with no expression of the protein (Neg.) and a sample of purified protein (Pure) were loaded for comparison. The soluble fractions were used for FIDA measurements. FIGURE S4: Overview of the protocol for expression and purification of de novo designed proteins using heat lysis. ALFA‐tag binders were expressed and the cultures processed following this protocol. Samples before and after heat lysis were taken and loaded on SDS–PAGE gels to compare their purity. A detailed protocol for this procedure can be found in protocols.io. FIGURE S5: SDS–PAGE analysis of soluble and insoluble fractions after heat lysis for PSD95‐GK domain binders. E. coli cells expressing binders 1A‐6B were subjected to heat lysis and the proteins present in the soluble (Sol.) and insoluble (Insol.) fractions visualized by SDS–PAGE. Cells with no expression of the protein (Neg.) and a sample of purified protein (Pure) were loaded for comparison. The soluble fractions were used for FIDA measurements. FIGURE S6: Titration curves of AF488‐labeled SH3‐GK with purified GK‐domain binders. Apparent R h of AF488‐labeled SH3‐GK (80 nM) as a function of binder conce [file PRO-34-e70286-s001.docx]

**Supplementary material**

**Screening *de novo* designed protein binders in unpurified lysate using flow induced dispersion analysis**

Francisca Pinheiro^1-3^, Jan S. Nowak^1^, Elena Zueva^1^, Emily C. Pheasant^1-3^, Ida Kjærsgaard Grene^1-3^, Vili Lampinen^1-3^, Magnus Kjaergaard^1-3^

^1^Department of Molecular Biology and Genetics, Aarhus University, Universitetsbyen 81, 8000 Aarhus C, Denmark

^2^Center for Proteins in Memory – PROMEMO, Danish National Research Foundation, 8000 Aarhus C

^3^The Danish Research Institute for Translational Neuroscience (DANDRITE), Aarhus University, Universitetsbyen 81, 8000 Aarhus C, Denmark

^*^For correspondence: Magnus Kjaergaard, magnus@mbg.au.dk


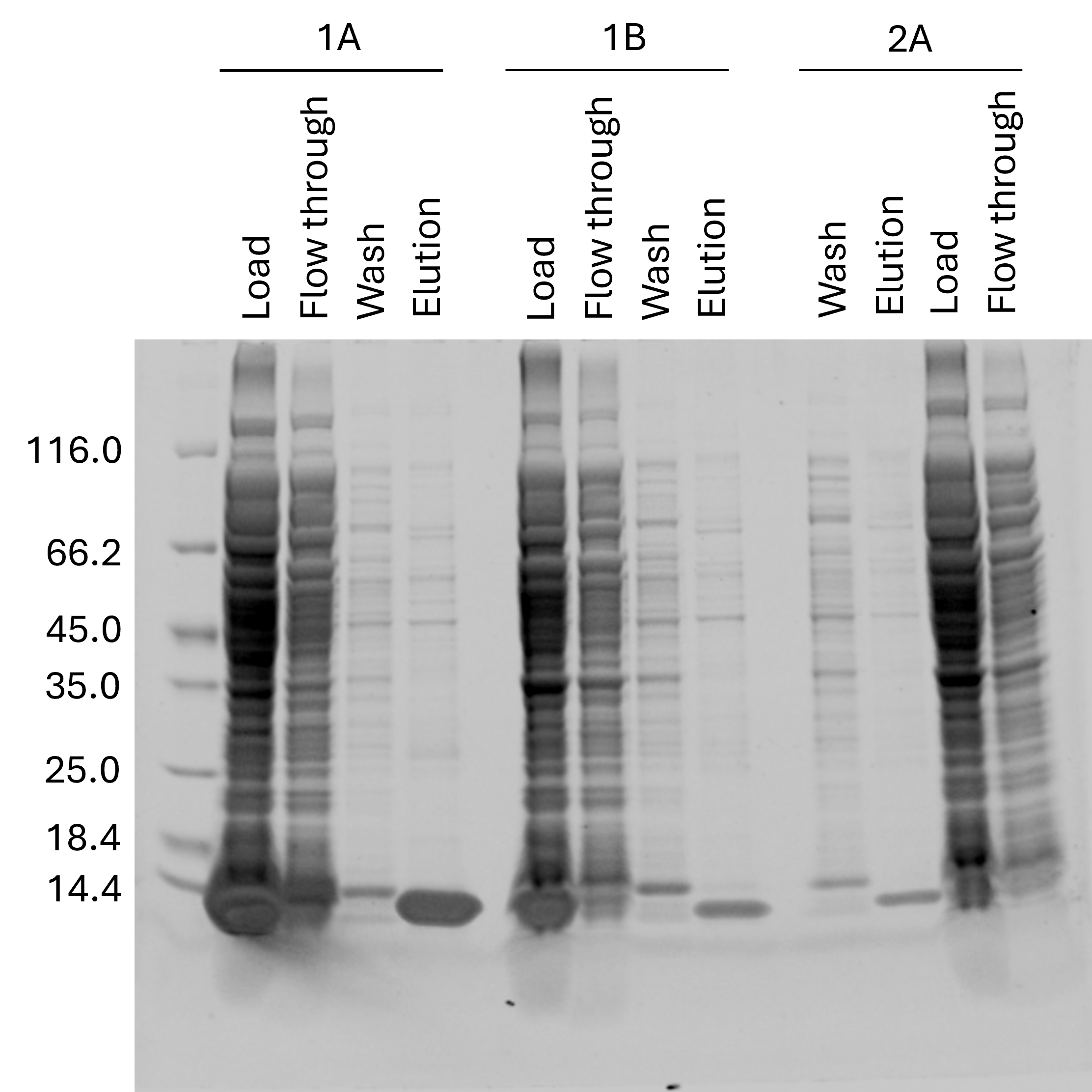


**Fig. S1. Purification of ALFA-tag binders.** Representative SDS-PAGE illustrating the Ni-NTA purification of ALFA-tag binders.


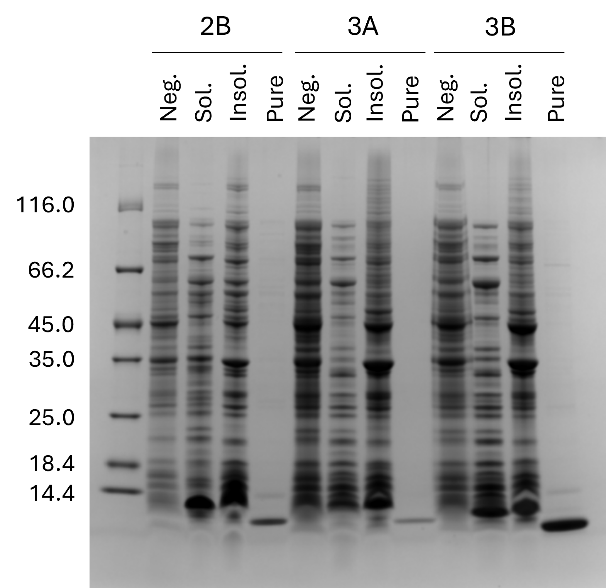


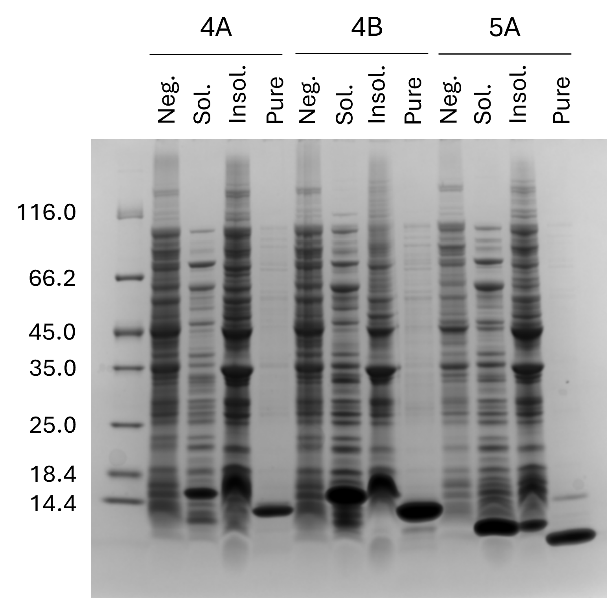


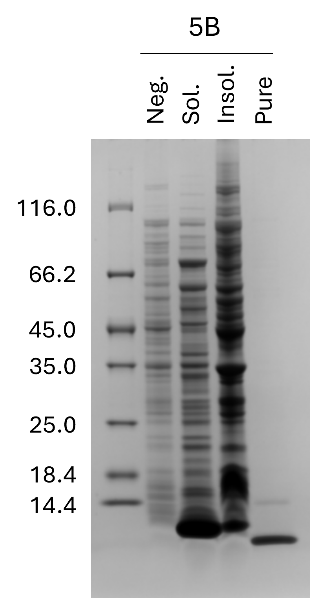


**Fig. S2. SDS-PAGE analysis of soluble and insoluble fractions after chemical lysis for ALFA-tag binders*.*** *E.coli* cells expressing binders 2B-5B were lysed with BPER reagent and the proteins present in the soluble (Sol.) and insoluble (Insol.) fractions visualized by SDS-PAGE. Cells with no expression of the protein (Neg.) and a sample of purified protein (Pure) were loaded for comparison. The soluble fractions were used for FIDA measurements.


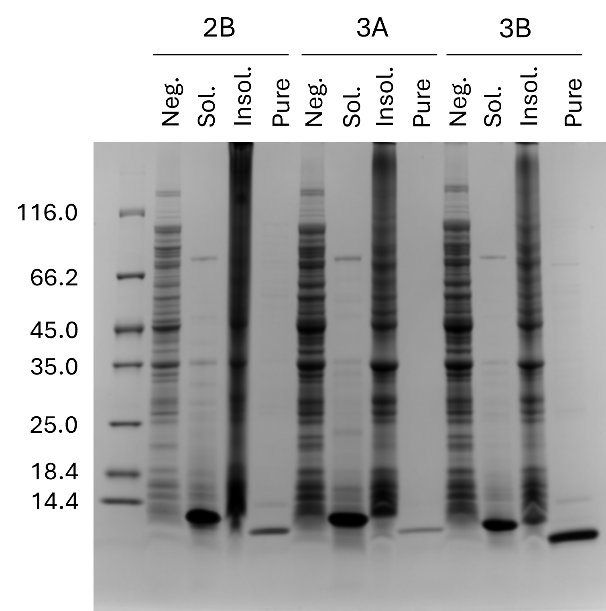


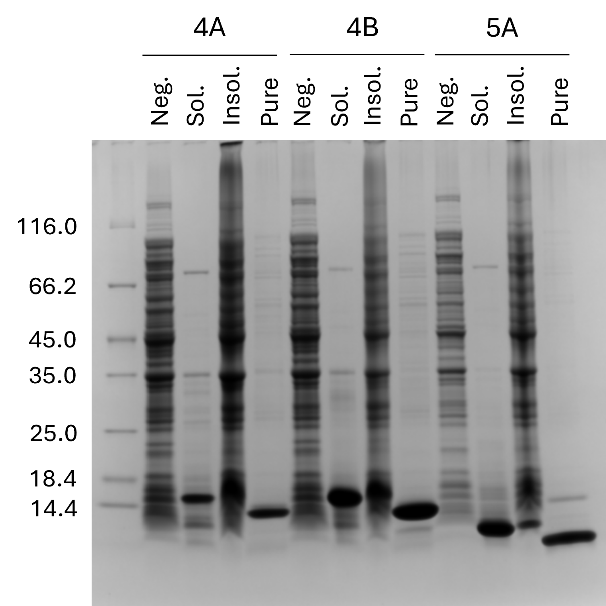


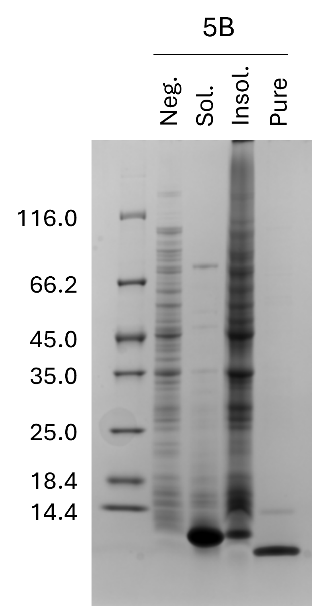


**Fig. S3. SDS-PAGE analysis of soluble and insoluble fractions after heat lysis for ALFA-tag binders*.*** *E.coli* cells expressing binders 2B-5B were subjected to heat lysis and the proteins present in the soluble (Sol.) and insoluble (Insol.) fractions visualized by SDS-PAGE. Cells with no expression of the protein (Neg.) and a sample of purified protein (Pure) were loaded for comparison. The soluble fractions were used for FIDA measurements.


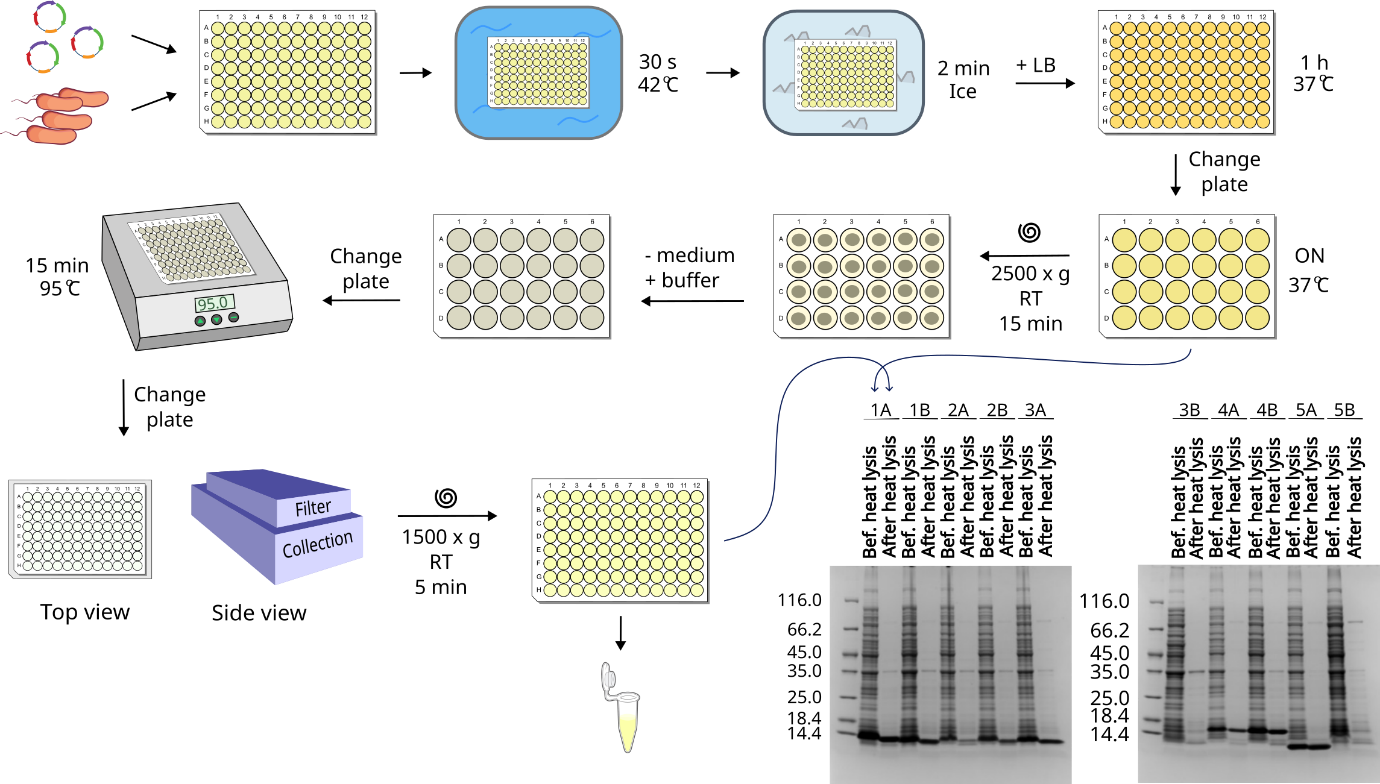


**Fig. S4. Overview of the protocol for expression and purification of *de novo* designed proteins using heat lysis.** ALFA-tag binders were expressed and the cultures processed following this protocol. Samples before and after heat lysis were taken and loaded on SDS-PAGE gels to compare their purity. A detailed protocol for this procedure can be found in protocols.io.


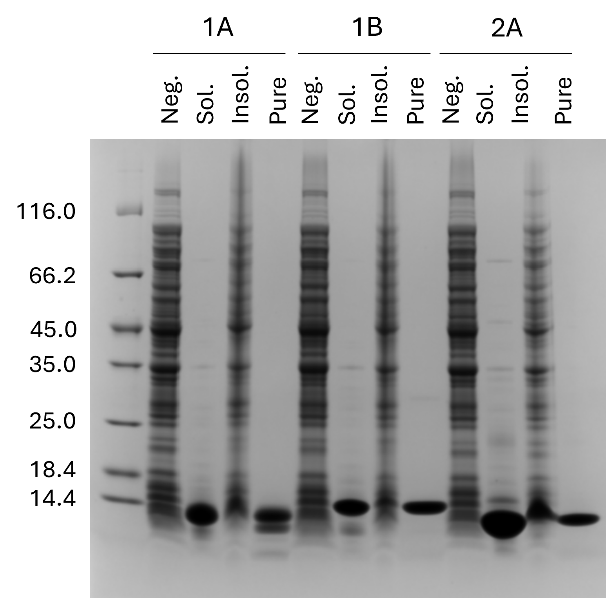

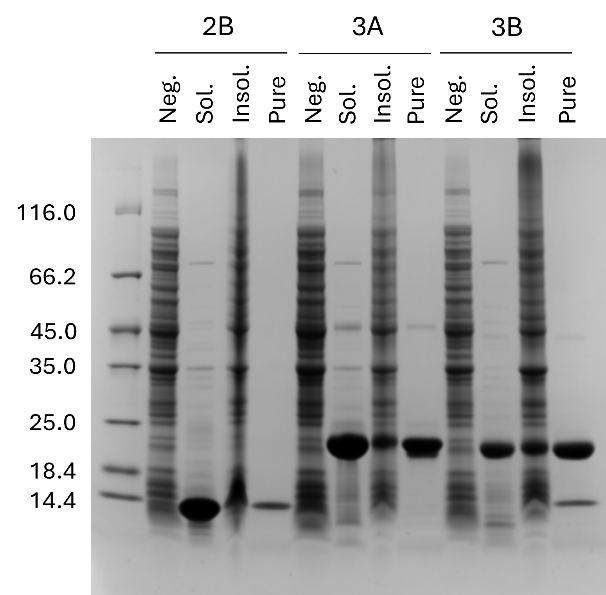


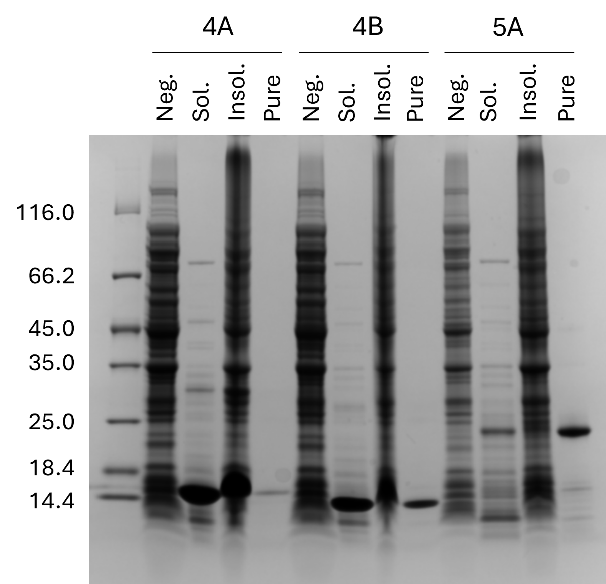

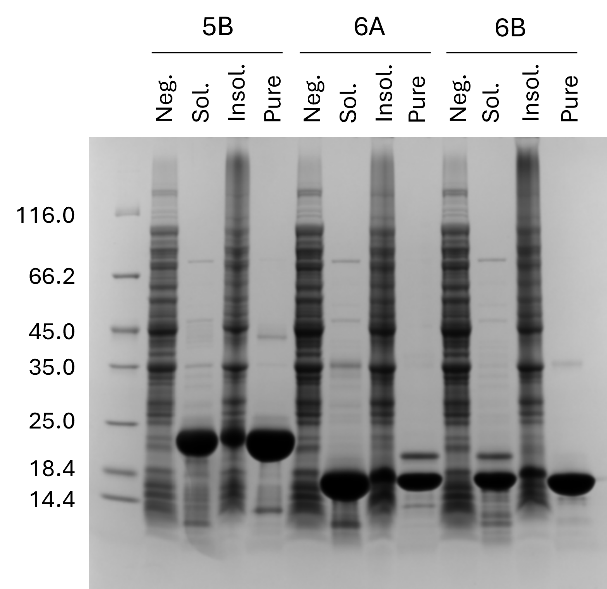


**Fig. S5. SDS-PAGE analysis of soluble and insoluble fractions after heat lysis for PSD95-GK domain binders*.*** *E.coli* cells expressing binders 1A-6B were subjected to heat lysis and the proteins present in the soluble (Sol.) and insoluble (Insol.) fractions visualized by SDS-PAGE. Cells with no expression of the protein (Neg.) and a sample of purified protein (Pure) were loaded for comparison. The soluble fractions were used for FIDA measurements.


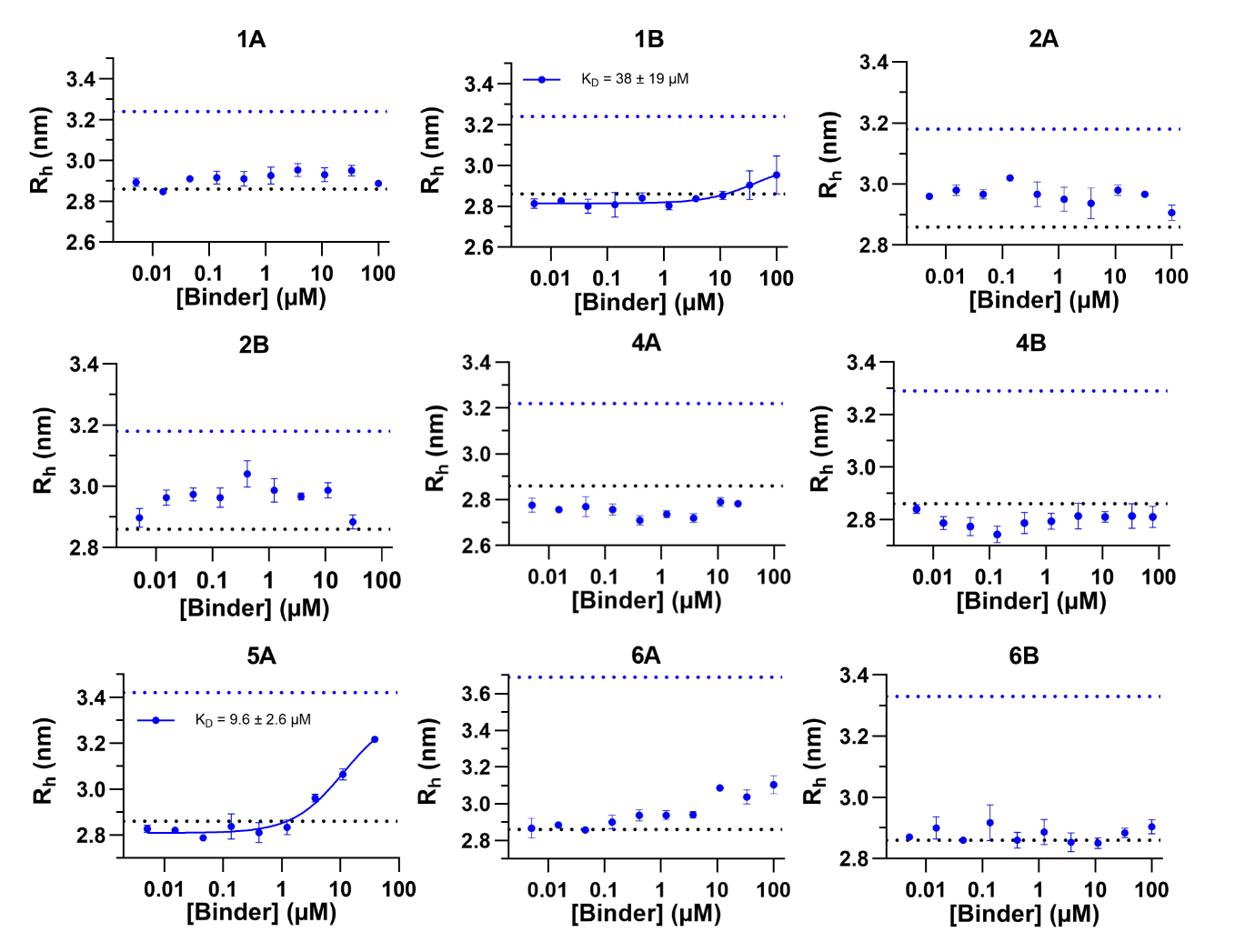


**Fig. S6. Titration curves of AF488-labelled SH3-GK with purified GK-domain binders.** Apparent R_h_ of AF488-labelled SH3-GK (80 nM) as a function of binder concentration. The R_h_ values represent mean ± SD (n = 3). In the case of binder 1B and 5A, the values could be fitted to a 1:1 binding isotherm, which is represented as a solid blue curve, allowing to calculate the K_D_. The black and blue dotted lines illustrate the predicted size of the SH3-GK domain alone and in complex with the different binders, respectively.

**Table S1. Amino acid sequence of the proteins purified in this study.**

Color coding:

6xHis-tag

Thrombin or TEV cleavage site

Binder

| **ALFA-tag binder 1A** |
| --- |
| MGSSHHHHHHSSGLVPRGSHMHMSEEFRKEIEERMERIRERLLRALETADLETLLALALEEVEELKEYYESLSEEEQEILFERLDEFFARLRAELAALTPELRAKALAALAALAEELA |
| **ALFA-tag binder 1B** |
| MGSSHHHHHHSSGLVPRGSHMHMSAAFREALRARAEARLEAFLEALRTKSVEELLALARAEVAELKELYASLSEEEQEILLEILDEAAERLRARLAELSPELRAKALEVLARLAEELA |
| **ALFA-tag binder 2A** |
| MGSSHHHHHHSSGLVPRGSHMHMSRREEIRARIEAELAAILRRIGARMESPELEPAIIALAARGYRELLALVEEDPELAERTRDARLAELEAELLALV |
| **ALFA-tag binder 2B** |
| MGSSHHHHHHSSGLVPRGSHMHMSRRDRIRAEVLARVAALLRRIGAEMADPALEPAVIRRAAAGLEELFALIDADPDAAAATLDARLAALEAELRALV |
| **ALFA-tag binder 3A** |
| MGSSHHHHHHSSGLVPRGSHMHMVTSPLELFLLLEEAFRELAEEEGDPERQERRRERLLELALELLGDDPRVERAARAARLLVDAERAGAPEEELAALRAALLAELA |
| **ALFA-tag binder 3B** |
| MGSSHHHHHHSSGLVPRGSHMHMVTSPLELYLLLYEAFRELAEEIGDEEEFEREAERLLALALEILGDDERVRLAYERARALVEAERAGLPAEELAALRAAVLAELA |
| **ALFA-tag binder 4A** |
| MGSSHHHHHHSSGLVPRGSHMHMSALERLRAAIEAEIKWKEEQAKKAEELGDKESAELFREIAELLETLYEFVELALEGEVERARRGFELALELALEAEREAAERLLEKFGNKEEAEEIRARAEASAEELRALFAELLA |
| **ALFA-tag binder 4B** |
| MGSSHHHHHHSSGLVPRGSHMHMSALEELKELLEKHIEWLKEKAKESKELGDEESAELFEEMAELTKTLFEFVELIEEGEVERGRRGFELALELLREAAKEAAELHKKKFGDEERAKEIIAYADDFTKRLTEKVEELIA |
| **ALFA-tag binder 5A** |
| MGSSHHHHHHSSGLVPRGSHMHMAEEELERLKEEILERVREVLESLSPEEREALERLSPEEALDRVLERMAELDEESRELAERARELLE |
| **ALFA-tag binder 5B** |
| MGSSHHHHHHSSGLVPRGSHMHMEEEERRRLEEEILEAVREALAALPPEERAALEELSPEEALDRVLELMAERDERSRALAEAARRLLE |
| **PSD-95 GK-domain binder 1A** |
| MGSSHHHHHHSSGLVPRGSHMSEVEEASLELDELIDQMERLLERAEEALEEAEKALKEGDLETAEKLLKVVEDHLRRFNSLYTRIGNELKKLPPELQAEYQKRIDELEERRLEILKRLAEL |
| **PSD-95 GK-domain binder 1B** |
| MGSSHHHHHHSSGLVPRGSHMEEVEEATEELNELLDQLERLLEIAEKNLEEAKKLLEEGDLESAERKLKIVEDHIRRFYSLYTRIGNELKKLPPEQQKIFNEKIDKLEERRLKILKELAEL |
| **PSD-95 GK-domain binder 2A** |
| MGSSHHHHHHSSGLVPRGSHMSAEKEAFLNGARVLSSALRKMLRERLKEYKETGSEEAAKYAEEVLKEMRELAEDLEKLGFEVEAVELKERIEEYEKELKKLKE |
| **PSD-95 GK-domain binder 2B** |
| MGSSHHHHHHSSGLVPRGSHMSAEKEAFYNGARVLWSALRKMARERLKEYKETGSKEAAEYLKRVLKEMRELAEDLRKLGFDVEATELEERIKEYEKELKELEK |
| **PSD-95 GK-domain binder 3A** |
| MGSSHHHHHHSSGLVPRGSHMFEARLQAALAAIGELADLYEDLYLETRKAYAEIKASKDWKERLKIVKELLKYVKEKMEAGLAREDEILVDAEAVLQEAYDRAPEEAESLESPASQIRGALLIKSSARHNIIELLHELKKLFAKDAEKPEAQEGKKLIEEIEKLLKGP |
| **PSD-95 GK-domain binder 3B** |
| MGSSHHHHHHSSGLVPRGSHMMQEELEAAKAAIGKLADTAEDLYLRTLEAYEKIKASTDWEEKLAITQELLAYVQAVMAAGYAERDAVLVDAEAVIQKAEEAAPEEAKSLESLTSEIKGALLIASSARSRIIELLHEMKKLFAKDAEHPAAQQGLELIDQILATLKGP |
| **PSD-95 GK-domain binder 4A** |
| MGSSHHHHHHSSGLVPRGSHMEKERREELFERYVRRAREIIERYKKIKPELSDDSEELRELVLEALDEVARAAILAGLKIYPLLIQRMLLVEKGDVLDEEALDYAERALERIEKKREEQRKEEE |
| **PSD-95 GK-domain binder 4B** |
| MGSSHHHHHHSSGLVPRGSHMAEAARRAALEAALAAARATLARLEALKPLLSDDSQELRDLAGDALDHAARAAILAGLDIHPLLVQRMLLLEKGDILVEEAVDYVRRVLERIEAKRAEQEAAAA |
| **PSD-95 GK-domain binder 5A** |
| MGSSHHHHHHSSGLVPRGSHMMIEEVEKKLKEMLEFSTKFMKELAKKFLEHLKKLVEKYGTEEIKKRAEKLKEFLDEFLNDFEDFLKRMFDAIIKYFKEGNIEKAAKVALDLARLFFRRAVRRVEEVKKEIEISTGFEGLVDAVVDYVMLVANTFAKNVDLEPLEIVEKMMEKLKEAAEKAEKELEKAMKKEEEEIEKKL |
| **PSD-95 GK-domain binder 5B** |
| MGSSHHHHHHSSGLVPRGSHMDIEEVKEKLKELLEKSLAFFKELADKVRAGLAAIVAETGTEEVARNAAILNEAIDELLNDIEDHLKRTIDAIIKYIEEGDIETAVKVALDLSRLFSRRAVRKVEEVRKESPLSTGFEITVDAIVDHIMRVANTFYKNKDLPPLERIEKVLEVLDESAKEAAARTDEGLAAYRAEVAARL |
| **PSD-95 GK-domain binder 6A** |
| MGSSHHHHHHSSGLVPRGSHMMEREEDLLLRRLREEGKEEEAEELELKLALELARSVLDLVEALAKALAKGDLQSAADIARLKMMSSDKTVHDLAEIFLRYMSALLEDPAAAGEVFTTDLEALLARYAPNPEVSALLQALLDAYRAAAGGPLATLAAALAAYAKWLEERAKELEKKLKE |
| **PSD-95 GK-domain binder 6B** |
| MGSSHHHHHHSSGLVPRGSHMKMRERDRLLRELREAGRLEEARRLELEEALELAEAVLPLVEALAKALEKGDYESALQIARLKMMSSNKTVHDIAEIFMRYMSALLQDPAKAAEVFTTALTELREKHAPNPEVAAILDALLEAYKAAAGKSPAELAAALRAYAEWLAARAAEARAELAA |
| **PSD-95 SH3-GK module** |
| MGSSHHHHHHSSGLVPRGSHMENLYFQSGSFYIRALFDYDKTKDCGFLSQALSFRFGDVLHVIDAGDEEWWQARRVHSDSETDDIGFIPSKRRVERREWSRLKAKDWGSSSGSQGREDSVLSYETVTQMEVHYARPIIILGPTKDRANDDLLSEFPDKFGSCVPHTTRPKREYEIDGRDYHFVSSREKMEKDIQAHKFIEAGQYNSHLYGTSVQSVREVAEQGKHCILDVSANAVRRLQAAHLHPIAIFIRPRSLENVLEINKRITEEQARKAFDRATKLEQEFTECFSAIVEGDSFEEIYHKVKRVIEDLSGPYIWVPARERL |

**Table S2. Purification yield of ALFA-tag and PSD-95 GK-domain binders.**

| **Binder** | **Yield (mg per litre of culture)** |
| --- | --- |
| ALFA-tag binder 1A | 148 |
| ALFA-tag binder 1B | 3 |
| ALFA-tag binder 2A | 11 |
| ALFA-tag binder 2B | 18 |
| ALFA-tag binder 3A | 15 |
| ALFA-tag binder 3B | 48 |
| ALFA-tag binder 4A | 39 |
| ALFA-tag binder 4B | 54 |
| ALFA-tag binder 5A | 45 |
| ALFA-tag binder 5B | 34 |
| PSD-95 GK-domain binder 1A | 327 |
| PSD-95 GK-domain binder 1B | 147 |
| PSD-95 GK-domain binder 2A | 181 |
| PSD-95 GK-domain binder 2B | 37 |
| PSD-95 GK-domain binder 3A | 114 |
| PSD-95 GK-domain binder 3B | 64 |
| PSD-95 GK-domain binder 4A | 33 |
| PSD-95 GK-domain binder 4B | 104 |
| PSD-95 GK-domain binder 5A | 18 |
| PSD-95 GK-domain binder 5B | 338 |
| PSD-95 GK-domain binder 6A | 164 |
| PSD-95 GK-domain binder 6B | 256 |
